# Concrete Condition Assessment Using Impact-Echo Method and Extreme Learning Machines

**DOI:** 10.3390/s16040447

**Published:** 2016-03-26

**Authors:** Jing-Kui Zhang, Weizhong Yan, De-Mi Cui

**Affiliations:** 1 Anhui and Huaihe River Institute of Hydraulic Research, No. 771 Zhihuai Road, Bengbo 233000, China; zjkah@163.com (J.-K.Z.); cdm@ahwrri.org.cn (D.-M.C.); 2 Machine Learning Lab, GE Global Research Center, Niskayuna, NY 12309, USA

**Keywords:** defect detection, extreme learning machine, feature extraction, machine learning, nondestructive testing, wavelet transform

## Abstract

The impact-echo (IE) method is a popular non-destructive testing (NDT) technique widely used for measuring the thickness of plate-like structures and for detecting certain defects inside concrete elements or structures. However, the IE method is not effective for full condition assessment (*i.e.*, defect detection, defect diagnosis, defect sizing and location), because the simple frequency spectrum analysis involved in the existing IE method is not sufficient to capture the IE signal patterns associated with different conditions. In this paper, we attempt to enhance the IE technique and enable it for full condition assessment of concrete elements by introducing advanced machine learning techniques for performing comprehensive analysis and pattern recognition of IE signals. Specifically, we use wavelet decomposition for extracting signatures or features out of the raw IE signals and apply extreme learning machine, one of the recently developed machine learning techniques, as classification models for full condition assessment. To validate the capabilities of the proposed method, we build a number of specimens with various types, sizes, and locations of defects and perform IE testing on these specimens in a lab environment. Based on analysis of the collected IE signals using the proposed machine learning based IE method, we demonstrate that the proposed method is effective in performing full condition assessment of concrete elements or structures.

## 1. Introduction

Early detection of internal defects of concrete structures and, more importantly, accurately identifying and characterizing the internal defects are crucial in ensuring the safety of concrete structures. Over the years, numerous non-destructive testing (NDT) methods have been developed and applied for detecting internal defects of concrete structures. These NDT methods include ultrasonic pulse, impact-echo, and ground penetrating radar technologies, to name a few. Several survey papers, for example, [1,2,3,4,5], provided an overview of different NDT methods. Among those different NDT technologies, the impact-echo (IE) method is the most popular one used for detecting cracks and delamination of concrete structures. The popularity of the IE method comes from its advantages of: (1) being truly nondestructive and non-invasive; (2) being suitable for single side detection; (3) being easy to use; (4) having relatively larger detection depth; and (5) and being less influenced by differences of concrete materials and structures [6,7,8].

**Figure 1:**
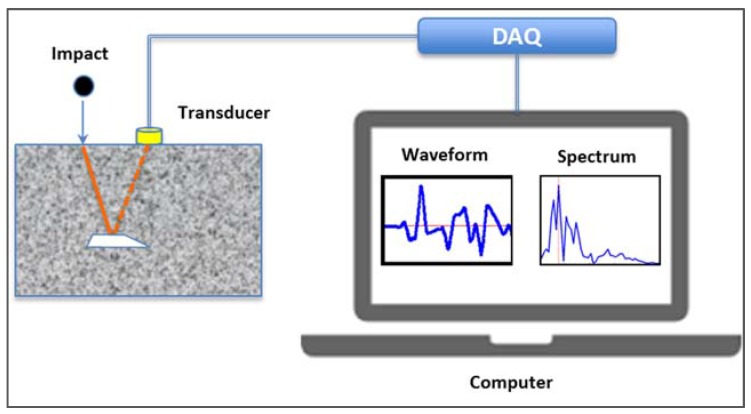
Schematic of impact-echo method.

The impact-echo method, introduced by Carino and Sansalone [9,10] in the 1980s, detects defects based on analysis of the reflection signals of impact-generated stress waves that propagate through the testing element/structure. Figure 1 shows a schematic of how the IE method works [9]. The success of the IE method calls for not only sensitive transducers and data acquisition (DAQ) systems to accurately measure the surface displacements caused by the reflections of the waves, but also intelligent data analysis and interpretation of the recorded measurements (waveforms). In traditional IE testing, data analysis and interpretation is merely to use the fast Fourier transform (FFT) technique to transform the time domain waveforms into frequency spectra and to infer the internal conditions by identifying the peak frequencies of the frequency spectrum. As a result, traditional IE is only effective for detecting certain defects (e.g., voids or delaminations) in plate like concrete structures, such as pavement slabs and bridge decks [8], but becomes less effective in detecting small, deep defects in non-plate like structures, e.g., concrete prismatic elements [11]. Most importantly, the traditional IE method has almost no capability of full condition assessment, *i.e.*, identifying the defect types and assessing the severity of defects. 

In recent years, efforts have been made to introduce more advanced data analysis techniques for better analyzing of IE signals. For example, in [12,13], ensemble empirical mode decomposition (EEMD) was used to decompose the IE signals into different spectra components for defect signal extraction. However, these improvements have been focused on defect detection; enabling the IE technique for full condition assessment has never been done, to the best of our knowledge.

Aiming for improving the IE technique in general and, more specifically and importantly, enabling it for full condition assessment, in this study, we attempt to introduce advanced machine learning techniques for strengthening the data analysis and interpolation of the IE technique. Specifically, we formulate the full condition assessment as a machine learning pattern classification problem. We use wavelet decomposition to extract salient signatures or features out of the raw IE signals and apply extreme learning machine (ELM) [14], a recently developed machine learning technique, as the classification model for full condition assessment. Our rationale is that the features extracted from the raw IE signals carry much richer information for capturing the IE signal patterns than the peak frequency shift used in the traditional IE method. Hence, by using advanced pattern recognition and classification on these high-dimensional features, not only are we able to detect concrete defects more accurately, we are also able to identify and characterize the internal defects in concrete structures. We validate our methodology by conducting analysis on the experimental data collected in the lab environment, and our validation results are very promising. To the best of our knowledge, machine learning techniques, especially wavelet based feature extraction and extreme learning machine, as a means of enhancing IE’s capabilities (improving defect detection and expanding defect identification) have not been studied before. Thus, our work in this paper could potentially contribute significantly in advancing NDT technologies in general and the IE method in particular. It is worth noting that our proposed methodology is general and potentially can be extended to other NDT methods, in addition to the IE method.

The reminder of the paper is organized as follows. In Section 2, a brief review of related work is provided. Section 3 gives detailed descriptions of the proposed full condition assessment methodology. In Section 4, we describe the experiments in detail. We present evaluation results in Section 5 and conclude our paper in Section 6.

## 2. Related Work

Since its inception in the 1980s [10], the IE method has been widely used for non-destructive evaluation of integrity and flaws of concrete elements and structures. Examples of such applications include bridge deck inspection [15], bridge deck crack detection [16], internal defect assessment of post-tensioned concrete slabs of high rise buildings [17], rebar corrosion damage detection [18], and shotcrete bonding state evaluation [19]. Over those years, continual improvements of the performance and the capabilities of the IE method have also been actively pursued. Among many of those improvements, we are particularly interested in research efforts that aim for improving the IE method through better interpretation of the IE signals. Broadly speaking, research efforts focusing on this part of improvements fall into two technology groups: *signal processing* and *machine learning*. One signal processing based improvement is to use time-frequency analysis as an alternative to the traditional FFT analysis of IE signals to take care of non-stationary of the IE signals. Abraham *et al.* [20] used short-time Fourier transform (STFT) to study the changing spectral properties of IE signals and they obtained an improved performance using time-frequency analysis of the IE signals. Empirical mode decomposition (EMD) [12,13], Hilbert–Huang transform (HHT) [21] have also been used for analysis of the IE signals. Wavelet transform is another time-frequency analysis technique that has been considered as the most advanced signal processing technique [22]. Compared to the traditional Fourier transform technique, wavelet transform has the capability of producing temporal and scale information simultaneously, thus is better suited for analyzing signals that are periodic, transient (or non-stationary), and noisy. As a result, wavelet transform has been widely used in numerous applications [23,24,25,26,27]. Recently, the wavelet analysis technique has also been adopted for IE signal analysis. For example, Luk *et al.* [28] used wavelet packet decomposition for impact acoustic non-destructive evaluation (NDE) of concrete slabs, where component power spectral density (PSD) patterns of some bases of wavelet packet decomposition (WPD) were used as inputs to the artificial neural network (ANN) for evaluating bounding quality. To enhance the Fourier spectrum of IE signals, Yeh and Liu [27] also applied wavelet transform. Specifically, they proposed two approaches: one was to combine the Fourier spectrum with the wavelet marginal spectrum in determining the echo peak, and another was to take the product of the two spectra. They used both numerical and experimental tests to validate the proposed approaches.

Machine learning techniques have been widely used for condition-based maintenance (CBM), structural health monitoring (SHM), and non-destructive evaluation (NDE). References [29,30,31] provide an in-depth overview of applications of machine learning techniques. Machine learning as a means of improving the IE method has been less explored, compared to signal processing. A few studies involve using different machine learning techniques, for example, artificial neural networks [28,29,30,31,32,33,34] and support vector machine (SVM) [35]. In [36], a Bayesian classifier was used for diagnosing internal defects of metal element using the IE technique. The features they used for classification were the spectrum of the IE signals captured by the seven accelerometers located at different surfaces of the test specimens. To reduce the feature dimensionality, they used principal component analysis (PCA).

Extreme learning machine (ELM), a special type of feed-forward neural networks, is one of the emerging machine learning techniques. Since its introduction by Huang in 2006 [14], ELM has been used in a wide range of applications, including computer vision [37], image processing [38], medication [39], and text understanding [40]. Recently, ELM has also been used for machinery fault detection and diagnosis, for example, [41,42,43]. Applying ELM to concrete condition assessment using the impact-echo method has not been done, to the best of our knowledge.

Based on our literature survey, a number of signal processing and machine learning techniques have been explored as a means of improving the data analysis and interpretation of the existing IE method, but most of the work was on improving the IE method in the context of concrete defect detection. To the best of our knowledge, using advanced machine learning techniques for enhancing the IE method for performing full condition assessment of concrete elements or structures has never been done.

## 3. The Proposed Methodology

**Figure 2:**
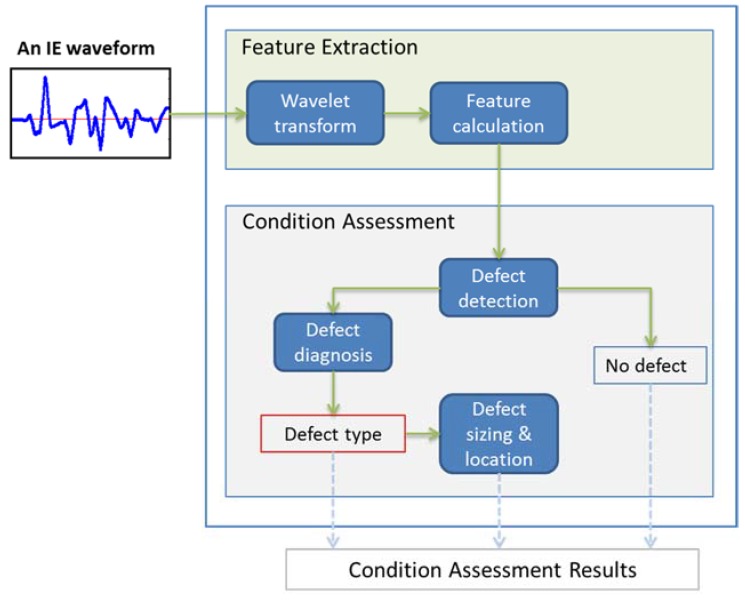
Overall flowchart of the proposed method.

Figure 2 illustrates the overall architecture of the proposed machine learning based full condition assessment framework. It consists of two primary analytics components, namely, (1) *feature extraction*, and (2) *full condition assessment*. We explain these two primary components in detail in the subsequent subsections.

### 3.1. Feature Extraction

Feature extraction is the process of extracting salient signatures or features from original sensor measurements or signals, which better represent the underlying problem to the predictive models. Like in the development of any other artificial intelligent systems, feature extraction is a critical task in designing our condition assessment system proposed. In literature, there are many feature extraction methods proposed for different applications. In this study, we adopt wavelet transform based feature extraction for extracting a number of salient features out of the IE signals. IE signals are generally transient in nature and contain different kinds of noise. Wavelet transform, an advanced signal processing technique, has the capability of producing temporal and scale information simultaneously, thus is better suited for analyzing signals that are periodic, transient (or non-stationary), and noisy [22]. Wavelet transform has been used for numerous applications [23,24,25,26,27] and for IE testing as well [26,27].

**Figure 3:**
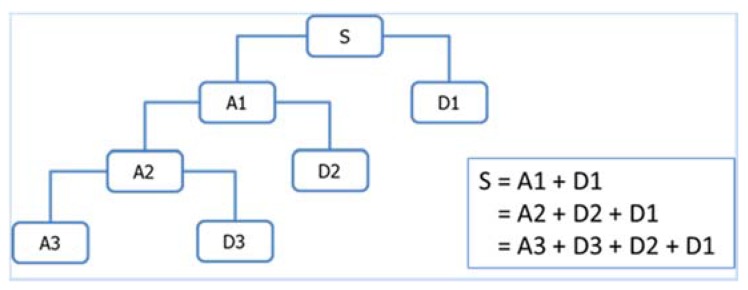
An example of 3-level wavelet decomposition.

Discrete wavelet transform decomposes a signal into a set of orthonormal bases that correspond to different time and frequency scales or resolutions [22]. At the first level of the decomposition, the original signal is decomposed into approximation and detail coefficients. The approximation coefficients are further decomposed into a second-level approximation and detail coefficients, and the process is repeated, which results in levels of approximations and details. The approximations are the high-scale, low-frequency components of the signal, while the details are the low-scale, high-frequency components. The multi-resolution wavelet decomposition results in the wavelet decomposition tree as shown in Figure 3.

**Figure 4:**
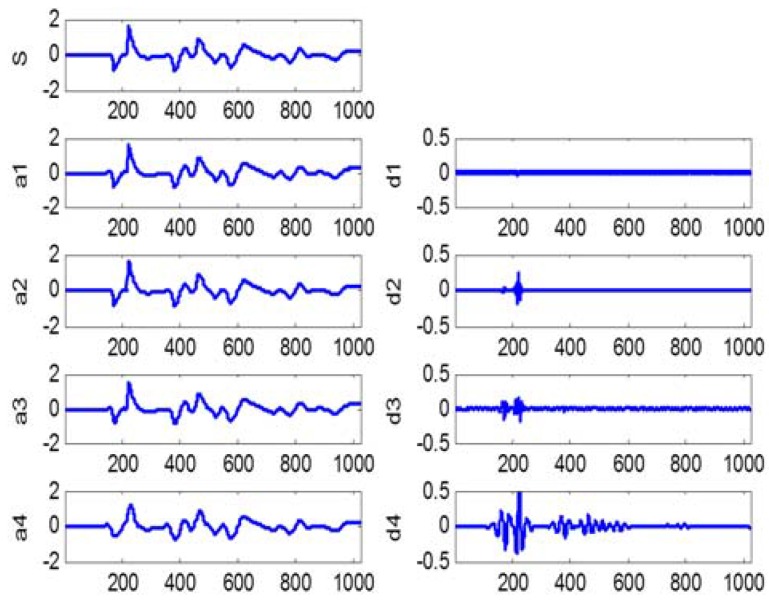
An example of wavelet decomposition of an impact-echo signal: s = original signal; a1…a4 are approximations; and d1…d4 are details.

In this study, we adopt 4-level wavelet decomposition for our impact-echo signals. Based on visual analysis of the impact-echo waveforms, we decide to use the 4th order “symlet” as the mother wavelet for wavelet analysis. Figure 4 shows the 4-level wavelet decompositions of a typical IE signal. We select A4, D4 and D3 as the bases for performing feature extraction. For each of the three selected bases, we apply feature calculation functions (defined below) on three domains (its wavelet coefficients, its reconstructed waveforms, and the spectrums of the reconstructed waveforms), respectively. We believe that features calculated on the three domains of each base signal can fully capture the characteristics of the base signal; and the combined features of all three selected bases (A4, D4, and D3) capture the significance of the original IE signal, while suppressing the noise, namely denoising, which is a well-known capability of wavelet decomposition. The 10 features extracted from each of the three selected bases are summarized in Table 1. For A4, we also calculate the depth frequency as an additional feature. All in all, we have a total of 31 (3 × 10 + 1) features. The feature calculation functions involved are further defined as follows.

Let $x_{n},\;n=1,2,\ldots ,N$ be the time domain signals and $\left[p_{i},\;f_{i}\right],\;i=1,2,\ldots ,\;M\;$ be its corresponding spectrum, where $p_{i}$ and $f_{i}$ are the amplitude and the frequency at $i^{th}$ frequency bin, respectively. The feature calculation functions are defined as follows.

Energy: $E=\sum_{i=1}^{N}x_{i}^{2}$; Total power: $TP=\sum_{i=1}^{M}p_{i}$; Mean power: MP=TPM,

(1) $$\text{1st spectral moment }(\text{centroid}):M_{1}=\sum_{i=1}^{M}p_{i}f_{i}/TP$$

(2) $$\text{2nd spectral moment }(\text{standard deviation}):M_{2}=\sqrt{\sum_{i=1}^{M}{\left(f_{i}-M_{1}\right)}^{2}\cdot p_{i}/TP}$$

(3) 3rd spectral moment (skewness): M3=∑i=1M(fi−M1)3⋅piM23⋅TP

(4) 4th spectral moment (kurtosis):  M4=∑i=1M(fi−M1)4⋅piM24⋅TP

### 3.2. Full Condition Assessment

As shown in Figure 2, our full condition assessment consists of defect detection, defect diagnosis, and defect sizing and location. Given an IE signal collected at a testing point, defect detection is to determine whether or not the test subject has any defects at the testing point. If a defect is found, then defect diagnosis, a process of determining the type of the defect, is triggered. In addition, defect sizing and location, to determine the extent or severity of the defect as well as the location (how deep along the wave propagation direction), is finally performed. From a machine learning modeling point of view, defect detection is a binary classification problem, while defect diagnosis, defect sizing and defect location can be formulated as multi-class classification problems, respectively, where the number of classes depends on how many defect types, defect sizes and defect locations are considered. Thus, designing the full condition assessment becomes designing a number of pattern classifiers.

In machine learning literature, there are a great number of classifiers available, ranging from traditional statistical methods to more modern methods, such as neural networks, support vector machines (SVMs), and random forests, to name a few. In this paper, we use extreme learning machine (ELM) as our classification models. ELM is a special type of feed-forward neural networks introduced by Huang *et al.* [14]. Numerous empirical studies, and recently some analytical studies, as well have shown that ELM is superior over SVM and other machine learning methods [44,45].

Unlike in other feed-forward neural networks where training the network involves finding all connection weights and bias, in ELM, connections between input and hidden neurons are randomly generated and fixed, that is, they do not need to be trained; thus training an ELM becomes finding connections between hidden and output neurons only, which is simply a linear least squares problem whose solution can be directly obtained by the generalized inverse of the hidden layer output matrix [14]. Because of such special design of the network, ELM training becomes very fast. ELM has been proven effective, especially when the number of training samples is small. The above-mentioned properties associated with ELM make ELM well suited for the concrete condition assessment concerned in this paper. Below is a brief description of how ELM works. Please refer to [14,44,45] for more details about ELMs.

Consider a set of *M* training samples, $\left(x_{i},y_{i}\right),\;x_{i}\in {\mathbb{R}}^{d},y_{i}\in \mathbb{R}$. Assume the number of hidden neurons of a single layer feedforward neural network (SLFN) is *N* and the activation function for each of the hidden neurons is *f.* Then, the outputs of the network can be expressed as

(5) $$f\left(x\right)=\overset{L}{\underset{i=1}{\sum }}\beta_{i}h_{i}\left(x\right)=h\left(x\right)\beta$$

where$\;h_{i}\left(x\right)=G\left(w_{i},b_{i},x\right),\;w_{i}\in {ℜ}^{M},\;b_{i}\in {ℜ}^{k},$ is the output of $i^{th}$ hidden neuron with respect to the input ***x***; G(w,b,x) is a nonlinear piecewise continuous function satisfying ELM universal approximation capability theorems [44]; $\beta_{i}\;$is the output weight vector between $i^{th}$ hidden neuron to the k≥1 output nodes. $h\left(x\right)=\left[h_{1}\left(x\right),\ldots ,h_{L}\left(x\right)\right]$ is a random feature map mapping the data from d-dimensional input space to the L-dimension random feature space (ELM feature space).

The objective of equality optimization constraints based ELM is to minimize both the training errors and the output weights, which can be written as [45]:

(6) $$\text{Minimize}:L_{p}=\frac{1}{2}{\left||\beta |\right|}^{2}+\frac{1}{2}C\sum_{i=1}^{N}\left||\xi_{i}|\right|{}^{2}\;\text{Subject to}:h\left(x_{i}\right)\beta =y_{i}^{T}-\xi_{i}^{T},\;i=1,\;\ldots ,\;N$$

where $\xi_{i}={\left[\xi_{i,1},\;\ldots ,\;\xi_{i,k}\right]}^{T}$ is the training error vector of the *k* output nodes with respect to the training sample $x_{i}$, and the constant C controls the tradeoff between the output weights and the training error.

The equivalent dual optimization objective function is:

(7) $$L_{d}=\frac{1}{2}{\left||\beta |\right|}^{2}+\frac{1}{2}C\overset{N}{\underset{i=1}{\sum }}\left||\xi_{i}|\right|{}^{2}-\overset{N}{\underset{i=1}{\sum }}\overset{k}{\underset{j=1}{\sum }}\alpha_{i,j}\left(h\left(x_{i}\right)\beta_{j}-y_{i,j}+\xi_{i,j}\right)$$

Based on the Karush–Kuhn–Tucker (KKT) condition, we can have the solutions for the ELM output function f(x) as follows (Refer to [45] for details):

(8) $$f\left(x\right)=h\left(x\right)\beta =h\left(x\right)H^{T}{\left(\frac{I}{C}+HH^{T}\right)}^{-1}Y,\text{when }N\text{ is not too big}$$

(9) $$\text{and }f\left(x\right)=h\left(x\right)\beta =h\left(x\right){\left(\frac{I}{C}+H^{T}H\right)}^{-1}H^{T}Y,\text{when }N\gg L$$

where ***H*** is the hidden layer output matrix.

(10) $$H=\left[\begin{array}{c} h\left(x_{1}\right)\\ \vdots \\ h\left(x_{N}\right) \end{array}\right]=\left[\begin{array}{ccc} h_{1}\left(x_{1}\right) & \ldots & h_{L}\left(x_{1}\right)\\ \vdots & \vdots & \vdots \\ h_{1}\left(x_{N}\right) & \ldots & h_{L}\left(x_{N}\right) \end{array}\right]$$

All in all, for the proposed full condition assessment methodology, we use four ELMs for defect detection, defect diagnosis, defect sizing and defect location, respectively. All of the four ELMs have the same input, *i.e.*, the 31 extracted features and only differ in the output. All ELMs use 1000 hidden neurons with sigmoidal as their activation function. The parameter, *C*, representing tradeoff between the output weights and the training error, is determined via cross-validation.

## 4. Experimental Details

To validate our proposed full condition assessment methodology, we perform lab experiments for collecting IE signals for data analysis. In this section, we provide details of our lab experiments.

### 4.1. Concrete Specimens for Testing

**Figure 5:**
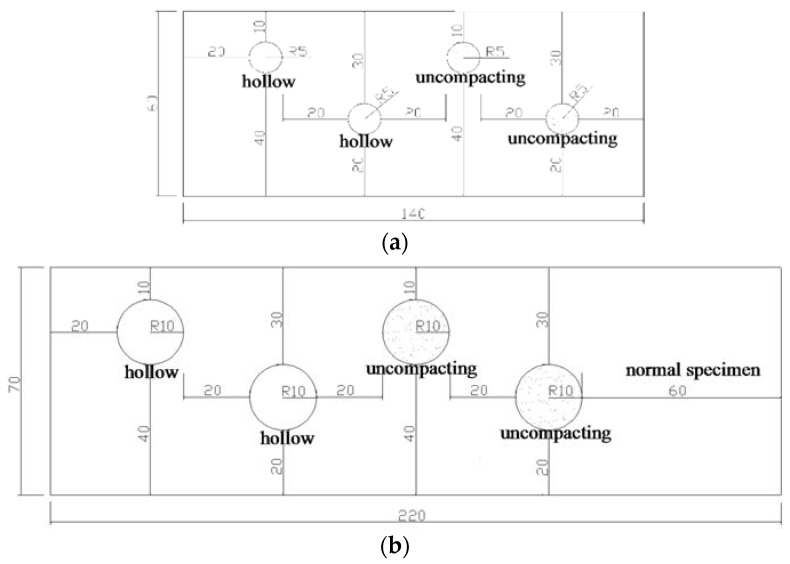
Cross-sectional views of specimens (Unit: cm): (**a**) 60 cm thick specimen with 10 cm defects; (**b**) 70 cm thick specimen with 20 cm defects.

**Figure 6:**
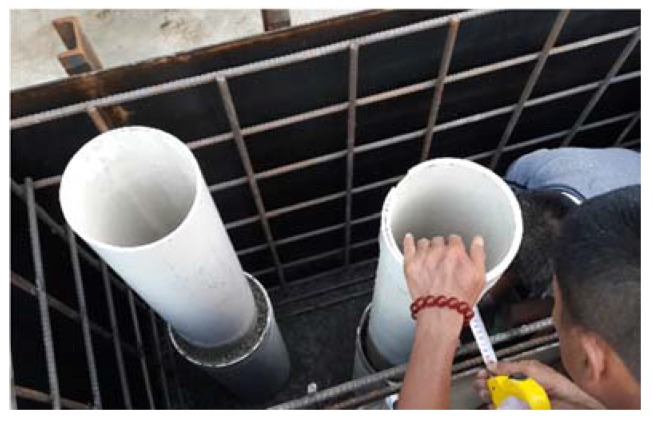
Cast-in hollow cylinders to represent defects.

Following the standard concrete casting and curing practice, we construct several reinforced concrete blocks with different sizes. The proportions of the concrete mix are shown in Table 2 and the 28-day compressive strength of the concrete is 30 MPa. The concrete blocks without defects are 40, 50, and 60 cm in thickness. Blocks containing defects are 150 cm tall and either 60 cm or 70 cm thick. The cross-sections for 60 and 70 cm thick blocks are shown in Figure 5a,b, respectively. Hollow cylinders are cast in to represent void defects (Figure 6). We can fill the hollow cylinders with water to represent water-filled void defects. We also have cylinder-shaped uncompacting defects as indicated in Figure 5, which gives us a total of three different defect types, voids, water-filled voids, and uncompacting defects. For each of the defect types, we consider two different defect sizes—10 and 20 cm in diameters. By performing the IE testing on both sides of the concrete blocks, we obtain four different defect locations (depths) of 10, 20, 30 and 40 cm, respectively, for each of the defects.

**Figure 7:**
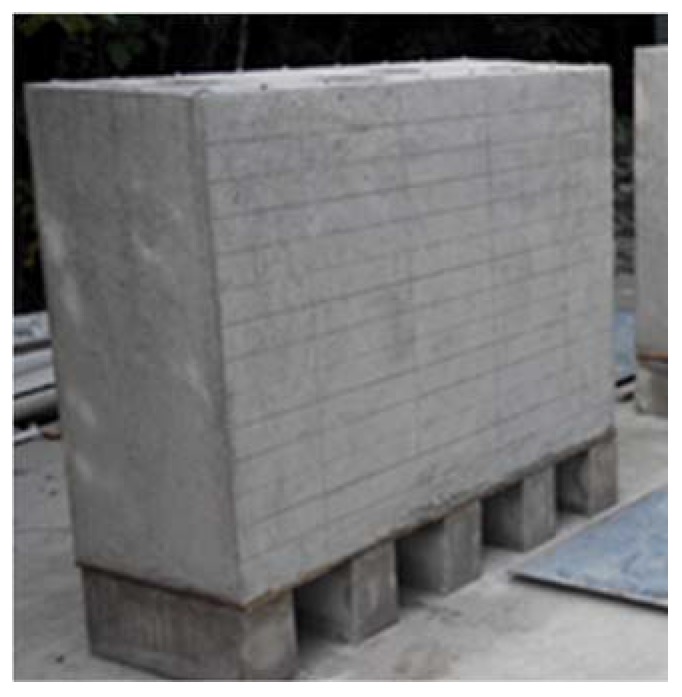
A specimen with grid lines marked.

**Figure 8:**
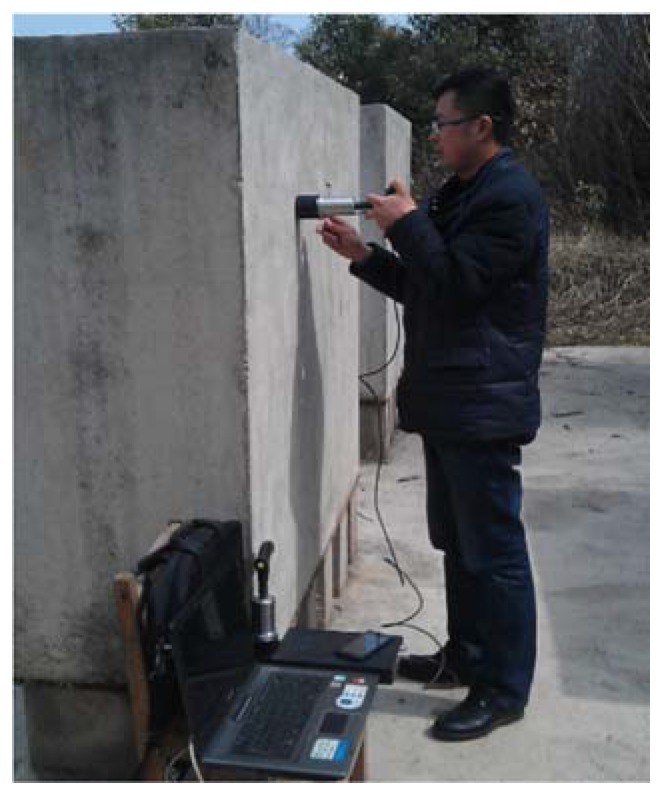
Conducting the IE testing.

To ensure the quality of the IE signals and efficiency of IE testing, the testing surfaces of specimens are polished carefully with sand papers and then rinsed with water. Both sides of the specimens are pre-marked with grid lines (Figure 7), where vertical lines are along the center axes of the four cylinders and horizontal lines are spaced at 10 cm, which give a total of 52 (=13 × 4) grid points or testing points per side. As the result, for each defect, we will have a total of 26 (=13 × 2) IE testing points, *i.e.*, 26 IE signals. Figure 8 illustrates how the IE testing is performed.

### 4.2. Testing Hardware 

**Figure 9:**
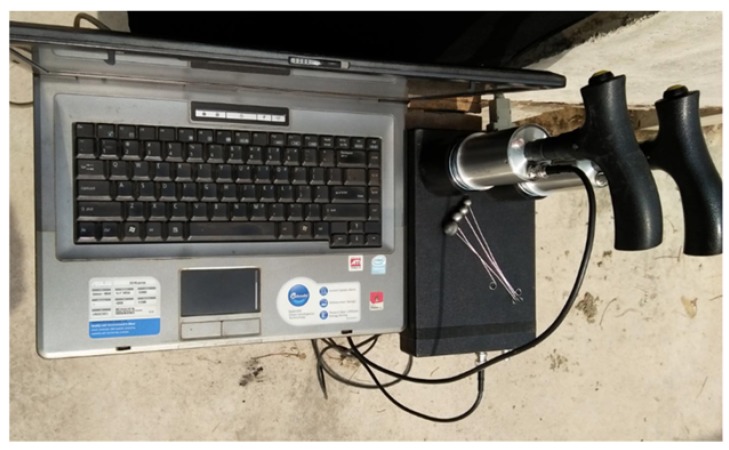
The hardware for IE testing.

The testing equipment we used is Impact-E type instrument made by the Impact-Echo Instruments, LLC, Ithaca, New York, USA [46] As shown in Figure 9, the test hardware consists of a set of steel impact balls, a handhold transducer unit, a DAQ system, and a computer with analysis software.

### 4.3. Test Parameter Setting

Setting of signal sampling frequency and sampling points is required to ensure that impact-echo signal with the maximum response frequency can be recorded on the basis of obtaining appropriate sampling resolution. In our testing, the sampling frequency is 500 kHz and the number of sampling points is 1024, based on the properties of our specimens.

Another critical test parameter is the size of the impact balls. As discussed in [6], different diameters of the impact balls will have different impact duration, or contact time; and the contact time is proportional to the amplitude of frequency components of the excited pulse and inversely proportional to the range of the frequencies of the pulse. In other words, ball diameter is inversely related to the maximum frequency that can be excited. Hence, we have to properly choose a steel ball such that the excited frequency range covers the frequency corresponding to the flaw depth. Given that our defect depths varies from 10 cm to 40 cm considered in this study, we have prepared a set of steel balls ranging in diameter from 6 mm to 18 mm.

## 5. Evaluation Results and Discussion

### 5.1. Evaluation Data Samples

Table 3 summarizes the IE signals collected in our test stand, where Type 1, Type 2, and Type 3 defects represent voids, water-filled voids, and un-compacting defects, respectively. An entry in the table indicates the number of impact-echo waveforms collected. As discussed in Section 4, for a typical defect type-size-location configuration, we performed 26 IE tests. However, for some configurations, we had to repeat the IE test twice (which gives us 52 IE tests) by experimenting with different impact ball sizes. Overall, a total of 858 IE signal (waveforms) were collected. While collecting these IE waveforms is already time-consuming, the number of samples is still relatively small in terms of classifier model training, especially for the defect sizing and the defect location models. In order to ensure models are properly trained, we follow the resampling techniques to increase the sample size [47]. Specifically, we generate replicas by adding a small amount of Gaussian noise to the features calculated from the collected samples. We perform robust cross-validation for model performance evaluation as discussed in the subsequent section, as a means of preventing model over-fitting.

### 5.2. Model Performance Measures and Performance Evaluation Method

This classification performance is typically summarized in a *confusion matrix* (also called *contingency table*) [48], where diagonal cells represent per-class classification accuracy and off-diagonal cells represent classification errors. For binary classification, cell (1, 2) represents false positive error (Type I errors, *i.e.*, *p*(false positive) = *p*(classified positive|negative) and cell (2, 1) represents false negative errors (Type II errors, *i.e.*, *p*(false negative) = *p*(classified negative|positive).

To assess classifier’s true performance for future unseen data, a popular 5-fold cross validation method [47] is used in this study. The *5-fold* cross-validation method divides the data set into five mutually exclusive subsets of approximately equal size. A classifier is then trained with four subsets and then tested on the remaining set. This is repeated five times and the final performance is the average of those of the five runs. Each example from the data set is used exactly once in a test set, and four times in a training set. Test sets generated by *5-fold* cross validation are independent because each instance is included in only one test set. In addition, all data analysis tasks, *i.e.*, data preprocessing, wavelet transform, and classifier modeling, are performed in a Matlab^®^ environment.

### 5.3. Results

#### 5.3.1. Defect Detection

For defect detection, we combine together all normal samples in Table 3a and label them as the “normal class”; and combine all defect samples in Table 3b together and label them as the “defect class”. Such labeled data are then used for training and validating the binary classification model. Table 4a shows the confusion matrix of the defect detection model, based on 5-fold cross-validation. From Table 4a, one can see that our defect detection model has a true positive rate (TPR) of 100.0% and a false positive rate (FPR) of 0.87%, respectively, which is an exceptionally good classification performance. For comparison, we also show the defect detection results (Table 4b) by using the traditional IE method, *i.e.*, spectral analysis. It is clear that our proposed method outperforms significantly the traditional method. The low detection rate (72.2%) of the traditional method is partially due to the fact that our 10 cm defect size is too small with respect to the defect depth of 40 cm. Based on Sansalone and Street [6], traditional IE spectral analysis is not effective when the defect size-to-depth ratio is less than 0.3. As shown in Table 3, there are about 1/8 of testing samples that have defect size of 10 cm and defect depth of 40 cm, which has the defect size-to-depth ratio of 0.25 (=10/40), much less than 0.3. The comparison results indicate that the proposed method is more effective than the traditional IE method in detecting small and deep defects.

#### 5.3.2. Defect Diagnosis

For defect diagnosis, we combine all testing samples under each of the three defect types, respectively, thus form a 3-class classification problem. The numbers of samples for the three defect types are 338, 208, and 208, respectively. The confusion matrix of the defect diagnosis model is shown in Table 5, which demonstrates that our fault diagnosis model can generally diagnose the three different faults well (with an overall classification accuracy of approximately 99%).

#### 5.3.3. Defect Sizing

Defect sizing is performed separately for each of the three defect types, that is, three different defect sizing models. Since we only collected signals for two different defect sizes, *i.e.*, 10 and 20 cm, we formulate our defect sizing as a binary classification problem, that is, to decide a defect is 10 cm (representing small defects) or 20 cm (representing big defects). In the future, we can expand our system to cover more levels of defect sizes. Table 6, Table 7 and Table 8 are the confusion matrices for differentiating sizes of Types 1, 2, and 3 defects, respectively. As can be seen from the three tables, our system can differentiate small (10 cm) and large (20 cm) defects well, especially for Type 2 (water filled voids) and Type 3 (uncompacting) defects.

#### 5.3.4. Defect Location

Defect location estimation is performed for different defect sizes as well as for different defect types, that is, we have six different defect location estimation models. Once again, we formulate our defect location estimation as a 4-class classification problem, and the four classes represent four different depths, *i.e.*, 10, 20, 30, and 40 cm. Table 9, Table 10 and Table 11 summarize the classification performance represented in confusion matrices for the six defect location estimation models. From these tables, one can see that except for small Type 1 defects (Table 9a), our defect location estimation models generally estimate defect locations well. Generally speaking, defect location estimation models perform better for larger (20 cm) defects, as indicated in Table 9b, Table 10b, and Table 11b, than smaller (10 cm) defects, as indicated in Table 9a, Table 10a, and Table 11a, which is consistent with our intuition. The poor performance of our model for small Type 1 defects may be partially due to the fact that about half of testing samples collected for Type 1 defects were done without using thin lead tape. Our initial experiments show that using thin lead tapes improve the quality (signal-to-noise ratio) of IE signals.

## 6. Conclusions

The impact-echo method, one of the many NDT techniques, has been popularly used for detecting internal defects of concrete elements. However, the IE method is only effective for detecting certain defects (voids or delaminations) in plate like concrete structures, and, most importantly, cannot be used for full condition assessment of concrete elements, mainly because its data analysis and interpretation involves the simple spectrum analysis and the simple thickness resonant frequency calculation. In this paper, we attempt to enhance the existing IE method through using advanced machine learning technologies for data analysis and interpretation of IE signals for more accurate defect detection and, most importantly, to enable the IE method for full condition assessment. Our machine learning based condition assessment system consists of two primary components, feature extraction and condition assessment.

Realizing that salient features that capture the characteristics of IE signals are the key to full condition assessment, in this paper, we adopt wavelet transform to decompose the IE signals into different bases and then extract a number of features from the selected bases. Such extracted features not only maximally capture the significance of the IE signals but also minimize the effect of noise, thus greatly increasing the signal-to-noise ratio. For full condition assessment, we apply extreme learning machine, a recently developed neural network technique, to classify the test object into different condition states based on the features extracted from the IE signals. By performing condition assessment on a number of test specimens built with different conditions in the lab environment, we are able to validate that the proposed framework can accurately and robustly assess the full health conditions of concrete elements.

To the best of our knowledge, enhancing the capabilities of the IE method from defect detection only to full condition assessment of concrete elements has never been performed before. We hope our study here can shed some light on enabling the IE method for full condition assessment. Our initial study reported in this paper is based on a limited number of specimens with a small number of defect types and sizes. In the future, we would like to do a more thorough study by using more test specimens. We also would like to validate our condition assessment system using real-world data, *i.e.*, the IE signals collected from real-world concrete elements.

## Figures and Tables

**Table 1:** Feature summary.

Domain	Feature Name	No.
wavelet coefficients	energy	1
reconstructed waveform	energy	2
spectrum of reconstructed waveform	total power	3
	mean power	4
	peak frequency	5
	mean frequency	6
	1st spectral moment	7
	2nd spectral moment	8
	3rd spectral moment	9
	4th spectral moment	10

**Table 2:** The proportions of the C30 concrete mix (kg/m^3^).

Cement	Medium Sand	Crushed Stone Aggregate	Coal Ash	Admixture	Water
360	708	1107	55	4.56	170

**Table:** (**a**)

Block Thickness	# of Sampling Points
40 cm	26
50 cm	26
60 cm	26
70 cm	26

**Table:** (**b**)

Defect Type->		Type 1 Defect		Type 2 Defect		Type 3 Defect	
Defect Size ->		**10 cm**	**20 cm**	**10 cm**	**20 cm**	**10 cm**	**20 cm**
Defect location	10 cm	52	26	26	26	26	26
	20 cm	52	26	26	26	26	26
	30 cm	52	26	26	26	26	26
	40 cm	52	52	26	26	26	26

**Table:** (**a**)

		PREDICTED	
		Normal	Fault
**TRUE**	**Normal**	99.13%	0.87%
	**Fault**	0.00%	100.00%

**Table:** (**b**)

		PREDICTED	
		Normal	Fault
**TRUE**	**Normal**	97.70%	2.30%
	**Fault**	27.80%	72.20%

**Table 5:** Confusion matrix of the defect diagnosis model.

		Predicted		
		Type 1	Type 2	Type 3
**True**	**Type 1**	98.31%	1.41%	0.28%
	**Type 2**	2.12%	97.71%	0.17%
	**Type 3**	0.16%	0.64%	99.20%

**Table 6:** Sizing for Type 1 defects (voids).

		Predicted Defect Sizes	
		10 cm	20 cm
**True defect sizes**	**10 cm**	99.02%	0.98%
	**20 cm**	0.52%	99.48%

**Table 7:** Sizing for Type 2 defects (water-filled voids).

		Predicted Defect Sizes	
		10 cm	20 cm
**True defect sizes**	**10 cm**	100.0%	0.0%
	**20 cm**	0.0%	100.0%

**Table 8:** Sizing for Type 3 defects (uncompacting).

		Predicted Defect Sizes	
		10 cm	20 cm
**True defect sizes**	**10 cm**	100.0%	0.0%
	**20 cm**	0.0%	100.0%

**Table:** (**a**)

		Predicted Defect Location			
		10 cm	20 cm	30 cm	40 cm
**True defect location**	**10 cm**	87.25	5.88	1.96	4.90
	**20 cm**	4.29	88.10	2.86	4.76
	**30 cm**	0.00	0.52	88.54	10.94
	**40 cm**	7.41	0.93	10.19	81.48

**Table:** (**b**)

		Predicted Defect Location			
		10 cm	20 cm	30 cm	40 cm
**True defect location**	**10 cm**	98.67	0.00	0.00	1.33
	**20 cm**	0.00	100.00	0.00	0.00
	**30 cm**	0.00	0.00	98.00	2.00
	**40 cm**	0.64	0.00	0.96	98.40

**Table:** (**a**)

		Predicted Defect Location			
		10 cm	20 cm	30 cm	40 cm
**True defect location**	**10 cm**	97.92	1.39	0.00	0.69
	**20 cm**	0.64	98.08	0.00	1.28
	**30 cm**	0.00	0.00	99.36	0.64
	**40 cm**	0.64	2.56	0.64	96.15

**Table:** (**b**)

		Predicted Defect Location			
		10 cm	20 cm	30 cm	40 cm
**True defect location**	**10 cm**	100.00	0.00	0.00	0.00
	**20 cm**	0.00	100.00	0.00	0.00
	**30 cm**	0.00	0.00	100.00	0.00
	**40 cm**	0.00	0.00	0.00	100.00

**Table:** (**a**)

		Predicted Defect Location			
		10 cm	20 cm	30 cm	40 cm
**True defect location**	**10 cm**	96.79	1.92	1.28	0.00
	**20 cm**	1.28	98.72	0.00	0.00
	**30 cm**	0.00	0.00	96.15	3.85
	**40 cm**	0.00	0.00	5.13	94.87

**Table:** (**b**)

		Predicted Defect Location			
		10 cm	20 cm	30 cm	40 cm
**True defect location**	**10 cm**	97.44	0.00	2.56	0.00
	**20 cm**	1.28	98.72	0.00	0.00
	**30 cm**	0.00	0.00	98.72	1.28
	**40 cm**	0.00	0.00	0.00	100.00

## Abbreviations

The following abbreviations are used in this manuscript:

ELM	Extreme learning machine
IE	Impact-echo
NDT/NDE	Non-destructive test/evaluation
